# Allergic Contact Dermatitis in Refractory Brick Production Worker: A Case Report

**DOI:** 10.7759/cureus.33732

**Published:** 2023-01-13

**Authors:** Negin Kassiri, Sahand Nikasa, Anita Aminizade, Yasser Labbafinejad

**Affiliations:** 1 Occupational Medicine, Iran University of Medical Sciences, Tehran, IRN; 2 Occupational Medicine, Iran university of medical science, Tehran, IRN

**Keywords:** allergic contact dermatitis (acd), refractory brick, workplace, exposure, chromium

## Abstract

In this study, we investigated a 42-year-old man working in a refractory brick (RB) production line who had allergic contact dermatitis (ACD) due to skin exposure to chromium (Cr). He had visited a dermatologist several times over a five-month period and although he had been medically treated, the symptoms reappeared after he returned to work and resumed exposure. Finally, with the announcement of the definite diagnosis of ACD through a patch test, it was decided to exclude him from exposure, and after 20 days, the symptoms went through the recovery process. No new recurring episodes were reported during the six-month follow-up period.

## Introduction

The refractory brick (RB) is a structure with high-temperature resistance and is used for industrial and smelting furnaces. The iron and steel industries are the largest customers of RB products in Iran. Refractory materials are based on six major oxides: silicon dioxide (SiO_2_), zirconium dioxide (ZrO_2_), magnesium oxide (MgO), calcium oxide (CaO), chromium oxide (Cr_2_O_3_), and aluminum oxide (Al_2_O_3_) [1]. The presence of Cr increases the flexibility of the brick and therefore its immunity against strains and thermal shocks [2]. The Cr is an intermediate metal that has a gray color, a glossy surface, and is hard and fragile. Chromium (Cr) is the major additive in the production of stainless steel, which causes its anti-corrosive properties. It also has a high polishing capability and excellent tarnish resistance. Individuals may be exposed via inhalation, gastrointestinal and cutaneous routes. If exposed acutely, it causes respiratory, ocular, and nasal irritation. If chronic exposure occurs, it can lead to ulcers and perforation of the nasal septum, coughing, shortness of breath, asthma, irritant, and allergic contact dermatitis (ACD) [3,4]. Allergic contact dermatitis (ACD) results from a cell-induced immune hypersensitivity reaction (type 4), which is less common in adults than irritant contact dermatitis [5]. The use of a patch test is the key method to diagnose the disease, which can be performed in different ways for suspicious substances. Temporary treatment is possible through skin cleansers, moisturizers, and topical corticosteroids, but the best thing is to keep the patient safe from exposure to allergenic compounds [6].

In this study, we examined a patient with ACD due to exposure to Cr compounds, natural history, diagnosis, treatment, and prognosis of the disease. Considering the low prevalence of ACD among occupational diseases and the widespread use of Cr in various industries, this case improved our knowledge in the process of follow-up and control measures of similar occupational and work-related diseases.

## Case presentation

**Figure 1:**
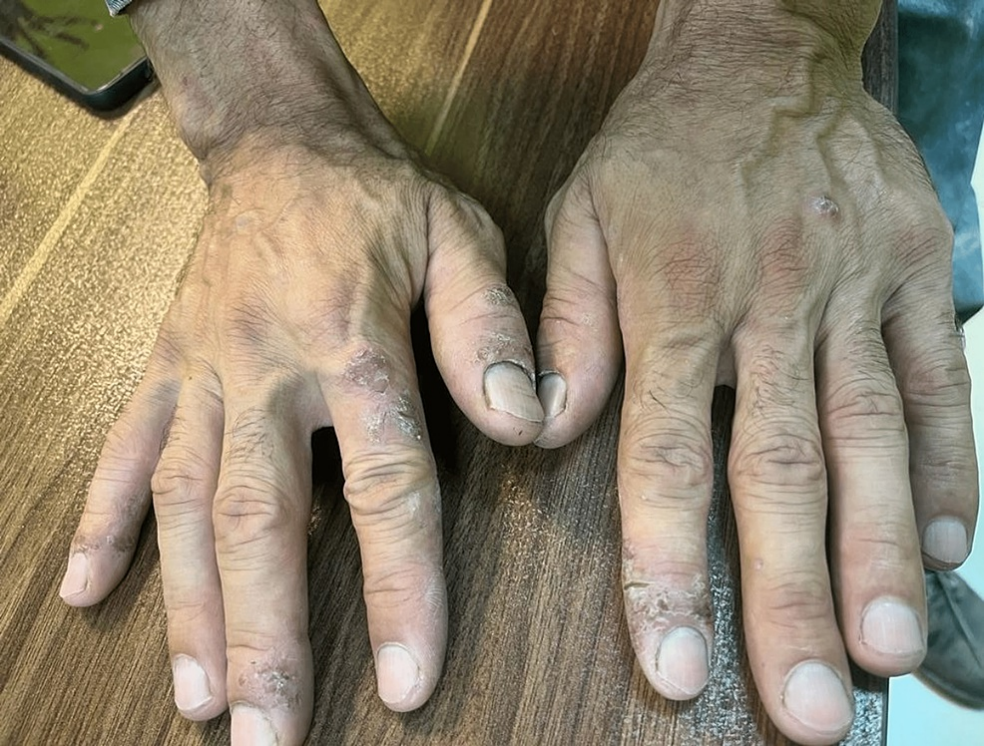
A 42-year-old man working in a refractory brick (RB) production line with allergic contact dermatitis (ACD) due to exposure to chromium oxide (Cr2O3).

A 42-year-old man working in the RB production line of an alloy steel company was referred to an occupational medicine specialist to assess his fitness for work. He has been working in this work unit for seven years. In the job hazard analysis, exposure to metal fumes, dust, infrared rays, heat, ergonomic exposures (including carrying heavy loads, standing for long periods, and improper body posture), shift work, and occupational stress was mentioned. According to the available material safety data sheets (MSDS), his main exposures were with Al_2_O_3_ and Cr_2_O_3_. He had no secondary employment. He was previously employed as an industrial cleaner. During the first few years of working in the production line of RB, he did not have any problems until the last five months; he had itchy papulovesicular lesions on the dorsum of both hands, and the areas between the fingers. The skin of the hands was dry, thick, and fissured (Figure 1).

Based on the appearance of lesions, clinical symptoms, and occupational exposures, differential diagnoses such as irritant contact dermatitis, ACD, atopic dermatitis, erythrodermas, lichen planus, psoriasis, rosacea, stasis dermatitis, seborrheic dermatitis, and others were raised. During two dermatologist visits at an interval of two months, considering the most likely diagnosis which was irritant contact dermatitis, he was treated with a variety of corticosteroid and moisturizing ointments, and the use of two layers of cotton gloves was recommended. He mentioned that the effect of the medication was temporary, and when he returned to work, despite the protections, he had a recurrence of the lesions. He had not reported any history of other chronic diseases and no issues were found in reviews of other organ systems.

On the third visit to the dermatologist, in one of the training hospitals, the patch test was performed with suspicious compounds by the expert dermatologist. The upper part of the trunk was chosen for the test, the suspected substances remained on the skin for 48 hours, and after 96 hours the test result was read. The result was reported as "+2" for Cr_2_O_3_. The interpretation was "creating erythema, edema, and vesicles following a strong reaction." The dermatologist stated in his report that according to the type and location of lesions, history of symptoms from five months ago, lack of appropriate response to routine treatments, partial improvement of lesions in vacations, reports of MSDS, especially the presence of Cr_2_O_3_ in the workplace, and patch test results, the most likely diagnosis for him is ACD following exposure to Cr_2_O_3_.

**Figure 2:**
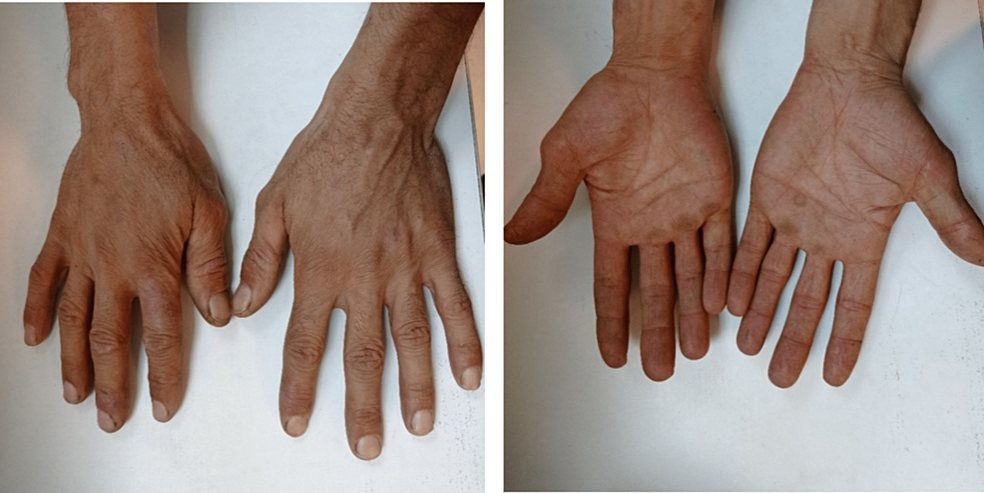
The healing process of skin lesions noted 20 days after exposure removal.

Considering the patient's type of exposure, his job, the impossibility of fully controlling the hazards, confirming the diagnosis of ACD, and the progressive nature of the disease, it was deemed unsuitable for the patient to continue working in this position, and the decision was made to remove him from the workplace. In consultation with the factory management, the worker was transferred to the product loading unit as a registration and loading operator. In his current job, he only has ergonomic issues. After 20 days of follow-up, the skin lesions went through the healing process and the symptoms decreased (Figure 2). During the six months of follow-up, no new episode of disease recurrence was observed.

## Discussion

Refractory brick is a type of ceramic porcelain that is made from refractory soils. There are various types of RB and depending on the type of material used in their compositions, they can withstand various temperature ranges. The ingredients in the corundum block are 90% to 95% Al_2_O_3_ and 5% to 10% Cr_2_O_3_. Chromium with atomic number 24 is a hard, shiny, and gray metal which has a high polish ability and boiling point. Cr is not malleable like other metals and is fragile. The highest value of this metal is due to its resistance to rust, erosion, and tarnishing. Adding it to steel prevents erosion and darkening [7]. Chromium, epoxy resin, biocides, aromatic substances, formaldehyde, rubber, and methacrylate are among the compounds that can cause an allergic reaction in case of skin contact [5]. A worker may come into contact with an allergen in the workplace months to years before the reaction occurs and suddenly experience symptoms of itching, redness, papule, and vesicle formation. Other complications include increased skin thickness, dryness, and the development of cracks.

In previous studies, several cases of ACD have been reported with Cr exposure in the cement, detergent [8], brassieres, [9], and cellular phones [10] industries. In a 2010 study by Lim et al., a case of ACD due to Cr exposure was reported in a female golfer following wearing of leather gloves that completely recovered after being advised to use Cr-free leather gloves [11]. In this study, we investigated ACD caused by exposure to Cr_2_O_3_ in RBs. Due to the low percentage of Cr_2_O_3_ used in RBs; no cases of ACD caused by it had been reported in our country.

This patient had reported frequent episodes of improvement and recurrence of clinical symptoms on the dorsum of the hands, and despite receiving routine topical treatments (moisturizing creams and corticosteroids), his recovery was not complete. The dermatologist had recommended removing exposures for his complete recovery. One of the main goals of the International Labor Organization (ILO) is to create opportunities for every man and woman to secure a decent job and increase the coverage and effectiveness of social protection for all. By removing a worker from the workplace, the issue of unemployment, poverty, and social damages are raised. For this reason, removing a worker from the workplace is one of the most difficult challenges faced by occupational medicine doctors. In this case, the chronic nature of the disease, the improving process during vacations, and the aggravation of the lesions when re-exposing, had a significant impact on the worker's presence in the workplace. Besides, hand involvement and oiliness of hands following the use of topical creams and ointments affected the worker's manual dexterity. The use of personal protective equipment such as gloves was also affected by lesions and local treatments in this person. Therefore, it seemed that the decision to remove the person from the workplace was an inevitable choice. Fortunately, by consulting with the factory management, we provided the opportunity for the worker to be re-employed in the same factory, but in an environment far from exposure to Cr_2_O_3_, and no new episodes were reported during the six months of follow-up.

## Conclusions

Refractory bricks have different compounds that can withstand various temperature ranges. Chromium is one of the additives in the production of RB, which can cause ACD in exposed workers. Allergic contact dermatitis results from a cell-induced immune hypersensitivity reaction (type 4), which is less common in adults than irritant contact dermatitis. Temporary treatment is possible through skin cleansers, moisturizers, and topical corticosteroids. Due to the allergic nature of the lesions, despite the temporary recovery, they appear again after the resumption of exposure. Therefore, removing exposure is the best decision for these patients.
